# Re-imagining the vulnerability and risk framing of parents with mental illness and their children

**DOI:** 10.3389/fpubh.2024.1373603

**Published:** 2024-05-01

**Authors:** Becca Allchin, Sophie Isobel

**Affiliations:** ^1^Mental Health Program, Eastern Health, Melbourne, VIC, Australia; ^2^School of Rural Health, Faculty of Medicine, Nursing & Health Sciences, Monash University, Clayton, VIC, Australia; ^3^University of Sydney, Faculty of Medicine and Health, Camperdown NSW, Australia

**Keywords:** parental mental illness, family mental health, framing, vulnerability, children of parents with a mental illness

## Abstract

To elicit compassion and communicate urgency to policy makers and governments, researchers and program developers have promoted a narrative of vulnerability and risk to frame the experience of families when parents have been diagnosed with mental illness. Developed within a western medicalised socio-cultural context, this frame has provided a focus on the need for prevention and early intervention in service responses while also unintentionally ‘othering’ these families and individualizing the ‘problem’. This frame has had some unintended consequences of seeing these families through a deficit-saturated lens that misses strengths and separates family members’ outcomes from each other. This paper raises questions about the continued fit of this frame and suggests a need to reimagine a new one.

## Introduction

Over the last 30 years researchers and service providers have sought to bring the attention of practitioners, policymakers and governments to families in which a parent experiences mental or substance use disorders. In this paper, we intentionally use the term ‘mental illness’ in line with the dominant language of the existing framing. However, we honor the important shifts that are occurring around how mental illness is understood and described, leading to the use of dynamic and humanistic terms, such as mental distress, mental health challenges, mental ill health, psychological distress and others. This present and emerging language is aligned with reimagining frames. We additionally acknowledge that the term ‘mental illness’ has historically had blurred boundaries and variously incorporated and excluded a range of conditions experienced by parents, including substance use disorders. Similarly, we refer to families and parents with acknowledgment that these relationships are socially defined and can mean different things to different people.

The body of work in the field of family mental illness has aimed to raise awareness of the prevalence and needs of parents with mental illness and their children. This has resulted in national and international efforts for change (1, 2) acknowledgment in law and policy, service development initiatives, the development of varied programs and a vast collection of literature bringing attention to the issues and a range of global research efforts documenting shifts and progress over time (3–8). Much of this work overwhelmingly presents these families, parents and children to be ‘vulnerable’ and ‘at risk’.

The vulnerability and risk frame positions children who have a parent with a mental illness as “among the most vulnerable in our communities” (9) (p. 350), at risk of mental illness, physical, social or psychological harm and vulnerable to further adversity as a result of their parents’ mental illness, subsequently requiring expert intervention “as early as possible to prevent future negative outcomes” (9) (p. 351). Framed in this way, urgency and priority are communicated for the purposes of recognizing the discrete experiences of this population, drawing attention to their needs and stimulating action. In this paper, building on long-standing calls (e.g., Gladstone, Boydell) (10), we suggest that despite the role it has played in raising awareness, there is a pressing need for overt reflection on the effects and challenges of this frame and building collective efforts to integrate a new one into academic, clinical and social discourse.

The concept of ‘frames’ has been constructed from the fields of linguistics, political science, sociology, and psychology. Framing relates to choices made about how information is presented and how these choices influence people’s attitudes, understandings, and actions (11). Frames are reinforced through words which use values, metaphors, tone or data to emphasize and de-emphasize patterns. Frames align to paradigms and are then reinforced through discourses, which shape how we see and think about people or populations and subsequently how society responds or supports them (12, 13). Discourses also influence which problems are identified (e.g., ‘parental mental illness’) and the solutions sought (e.g., ‘early intervention to mitigate risk’) and form the mental structures that shape our ideas and concepts to give us our understanding of reality (13).

## What underpins the vulnerability and risk frame

Frames exist in the context of wider society. In western society, the context of the risk and vulnerability frame is influenced, in part, by the intersecting influences of medical approaches to health and illness, neoliberal approaches to government and individualistic social structures. Governments across western countries largely drive policy agendas that reflect neoliberalist politics (14), there by reinforcing individualist constructs of health and wellbeing as personal choices, rather than complex interconnections between systems. This cultural framing of individualism has also led to a separate focus on individual family members; for example, parent mental health, child mental health and infant mental health are viewed as distinct streams which function often in isolation, and sometimes in competition (15). Individualist approaches also foster a preoccupation with interventions targeting specific behaviors or situations (15) with a focus of funneling resources to those ‘most at risk’ (16). This approach has been seen across the family mental health field, with increasing compartmentalisation and copyrighting of interventional models, with a seeming lack of reflection on their shared components, sustainability or equitable distribution.

Interventions which target vulnerable children, such as those with a parent with mental illness, are largely in place to reduce future burdens upon society from adults with complex needs or incapacity. In this way, children are framed as future adults who exist within a binary of ‘productive or unproductive’. To mediate this binary, discourses of risk and vulnerability also foster a concurrent discourse of resilience. Positioning some individuals or families as resilient places the agency and responsibility in the individual and distracts from wider government and social responsibilities. It also justifies more interventions and programs, including supporting identified ‘vulnerable’ individuals to cope with their conditions and circumstances through a depoliticised lens (17). Focusing on building individual resilience rather than reducing harm can also serve to justify cuts to systems like welfare and child protection (17).

Concurrently, western mental health systems are largely based around biomedical understandings of health and illness in which altered states or distress are understood as having a biological basis (18). While biomedical approaches have enabled a systemised way of studying mental distress and raised awareness and legitimacy of mental health as a component within health, they also inherently devalue the relational components of families, except as supportive or practical aides to individual treatment approaches. Subsequently within adult mental health services, parenting status remains underreported, and even within progressive models of care, families remain largely side-lined except when viewed through a lens of risk and vulnerability. To sustain the dominant paradigm of biomedical psychiatry and the associated frame of risk and vulnerability, research funding and service models and outcome approaches are prioritized which position mental illnesses as brain disorders requiring biological and pharmacological treatments to target imbalances and abnormalities. For children of parents with mental illness, this has resulted in research focusing primarily on identifying and responding to risks and vulnerability.

## Purposes the frame has served

The language and concepts of discourses shape the way problems are understood and the subsequent actions required. Discourses are productive, as Bacchi states ‘*Discourses accomplish things. They make things happen*’ (19) [p. 35]. The framing of families, parents and children as vulnerable, with children positioned to be ‘at risk’ of poor outcomes and intergenerational mental illness, has served a number of social, political and pragmatic purposes. It has led to an academic and clinical focus on identifying and articulating risks and mental health outcomes, advocating for families in which risk is highest and funding interventions to reduce vulnerability. It has also created legitimacy for families and children in the space of intergenerational intervention, advocated for prevention and early intervention within service paradigms and formed a shared language for services, clinicians, policy-makers and politicians to amplify urgency. It has also contributed to international action and momentum, generating prospective and collective knowledge, practice and policy to manage risk (20).

The discourse of infant and child mental health has created actionable directions through its focus on neuroscience, genetic vulnerability, recognizing the importance of the early years for lifelong health and development and emphasizing critical periods of intervention. It has also re-centered the primacy of attachment and family relationships as a foundation for development and increased awareness of the impacts of childhood adversity on development, leading to resource development and interventional models. Notions of risk and vulnerability in this way have created a sense of urgency which has enabled a focus on, and subsequent funding for, preventative approaches and early intervention.

The identification of particular groups of families, parents and children as ‘vulnerable’ has served to create a platform for connectedness to others with shared understanding. The creation of spaces that privilege a group based on their shared identity can assist in defining the self in relation to others (21). This fostering of group identity can help to mitigate stigma, decrease isolation and enable a sense of belonging. The frame has thus enabled opportunities to promote equity, focusing attention on the needs of families, parents and children, which in turn has provided the foundation for numerous peer support programs (22, 23) as well as other well-received and respected interventions.

Mental health awareness campaigns and the vulnerability and risk frame have led to increased service demand and sector justification. Foulkes and Andrews (24) propose that this relationship may be bi-directional, that is, increased rates of mental health problems drive increased awareness efforts, but the awareness efforts themselves then lead to increased reporting and experiencing of symptoms, a cycle they call prevalence inflation. Prevalence inflation serves important purposes in driving industry, justifying increased specialized services and funding and reducing stigma. In this way, the framing of children of parents with mental illness as vulnerable and needing interventions has allowed for activation of service responses to support families. In short, focusing on systematically identifying parents and children within mental health services, benevolently othering them based on their determined risk and vulnerability and then devising interventions delivered by experts to reduce their genetic load and prevent intergenerational mental illness has served an important role in activating and sustaining service responses. However, it also activates problematic ideas, values and understandings.

## Challenges caused by the frame

The frame of vulnerability and risk creates a narrowed view which overlooks families’ other circumstances, challenges, resources and strengths, positing that the illness of a parent is the primary ‘problem’ which then determines the family members’ individual and collective outcomes and needs. Without a broader lens of the social and structural determinants of health and wellbeing, the ideas for how to promote wellbeing are constricted to illness-related interventions or solutions (25). Not only does this strip the family of the opportunity to be seen and understood within their complexity, it also locates the problem at a family level, obscuring community, systems or structural solutions that are needed.

Positioning the parent’s illness as ‘the problem’ can separate family members’ outcomes, at times creating a false choice of parents’ needs versus children’s’ needs when one is prioritized or centered. For example, when children are at risk because of a parent’s illness or their own experiences are viewed as symptoms of vulnerability, individuals are unintentionally placed in opposition, distracting from the intertwined and bi-directional nature of familial experiences (26–28). In addition, the framing of risk and vulnerability can lead to parental shame and self-blame, undermining agency, self-efficacy and sense of confidence in parenting. The frame also makes parents with mental illness less likely to identify themselves in service systems or to seek support for themselves or their children, due to risk of judgment and scrutiny (29).

A deficit focus on parents, children and families within services and research leads to an over-emphasis on assessing risks, needs and shortfalls and creating problem-saturated formulations which can get in the way of providing effective support. For example, an evaluation of a Dutch family needs tool (30) identified that the safety and risk frame surrounding its use, led to a practice overly focused on ‘truth-finding’ about safety, rather than identifying needs for the provision of support. Thus, the frame influences how engagement occurs, creating relational suspiciousness, at times leading to assumptions of incapacity and creating environments where the parent, child or family feel defensive, undermining trust and the opportunity for working in collaboration. Safety and trust have long been known to be essential for therapeutic efficacy across disciplines or care modalities (31–33).

Embedded in the vulnerability and risk frame is a benevolent ‘othering’ of children and parents impacted by parental mental illness. Othering refers to dynamics and processes that engender exclusion based on group identities (34). While benevolent, all othering creates a binary (i.e., those with vulnerability and those without) promoting deviation from the norm which can dehumanize and pathologize difference. Even benevolent othering can invite an internalizing of vulnerability, when being “at risk” is no longer just about the probability of some hazard impacting on you; it is also about who you are as a person (35).

Despite children in families in which parents experience mental illness having diverse experiences and outcomes (23, 36), a vulnerability and risk frame promotes a clustering of experiences toward a binary of resilient or not. It encourages a simplification of stories to portray individuals as heroes who have overcome risks and adversities to become resilient, or victims of circumstances with an inevitability of poor outcomes. However, neither of these narratives do justice to understandings of resilience as a dynamic and interactional continuum that co-exists with adversity (37), nor the complexity and dynamic nature of families and circumstance which can both simultaneously promote and undermine wellbeing.

## Conclusion

The vulnerability and risk frame is influenced by the dominant paradigm of mental illness which underpins services, systems and the status quo. Scientists, academics and clinicians are educated within these paradigms and then function within the frame provided by them; they are then socialized into discourses, in ways so ingrained that people are unaware of their presence. The frame is reinforced by discourses which are linked to power, they are influenced by those in power and reinforcing it (20) Changing frames is therefore complex. In his seminal work on how paradigms shift, Kuhn (38) identifies that as evidence of discrepancies and challenges to the dominant paradigm accumulate, questions are asked of the accepted norm until a crisis occurs where the existing paradigm must be replaced by a new one. We posit that a crisis is occurring in the current paradigmatic positioning of family mental health and the associated frame that drives how parents, children and families are seen, understood and responded to.

Conflicts between recovery/wellbeing paradigms and illness/treatment paradigms in mental health care and services are widespread (39–41) as societal shifts challenge the dominant paradigm of mental illness. While new discourses are emerging and not yet integrated, it is timely for the field to examine the assumptions embedded within the paradigms and discourses that have created and sustained the vulnerability and risk frame for children and parents in families with parental experiences of mental ill health or distress. While acknowledging the purposes the frame has served, the discrepancies and challenges it creates demonstrate a need for open and overt discussion with all stakeholders to re-imagine new frames that are fit for purpose of the emerging paradigm. The re-imagining process needs to address inevitable concerns about what might be lost in shifting frames, such as how to measure efficacy, how to direct implementation and how to ensure children who need support are not being missed. Reimagining requires safe spaces to question positioning, assumptions, power and influence with openness to authentic partnership with those with lived and living experience as parents, children and families, attending to the dynamics of participation and power (42).

While there is no quick fix on the journey toward re-imaging and reframing understandings of family mental health in a way that maintains momentum, some key directions are clear. New frames need to honor people’s ability to act in their own lives while acknowledging the inequities in the systems and structures that limit their agency and autonomy. At a micro level this means new frames need to promote and privilege the voices and actions of those whose lives are most affected. At a meso and macro level this means holding individual and family wellbeing within a context of social justice and ecological health (43). This approach would support a deep understanding of the inequities inherent in the field and utilizing research for advocacy for change.

A new frame needs to shift the focus away from risk identification and vulnerability labeling, to identifying what people need to do well within their lives, families and context. Identifying needs at these micro, meso and macro level opens opportunities to develop solutions that honor the uniqueness of each situation and promote wellbeing rather than focusing on mitigating assumed ‘impacts’. A new frame needs to position people in their complexity. At an individual level this means exploring their strengths and challenges concurrently, not as opposing forces but as synergistic entities. People can be both vulnerable and resilient simultaneously. At a family level this means holding the outcomes of the family members together, rather than hierarchically positioning the outcome of one as the cause of another. A new frame should position everyone’s wellbeing as intertwined, with positive outcomes achieved when family members and communities are supported and empowered to promote the wellbeing of each other.

A new frame for working with and talking about families who experience mental distress or adversity would move beyond idealized solutions that come from the assumption that by systematically identifying, labeling and enacting formulaic actions, experiences can be avoided or prevented. Instead, a new frame needs to promote best outcomes for all involved as they find ways through the adversities all families face. Rather than directing actions as per a framework or model, a new frame requires repositioning assumptions, labeling and values about what it means to be a parent or child within a family experiencing adversity and why responses are diagnostically or categorically driven.

To progress the field of family mental health, we call for critical reflection on the frames that currently drive our research, practice and systems, reinforce assumptions about families and individuals and unintentionally cause harm. Existing frames have successfully raised awareness and urgency for the field, but in line with shifting paradigms of health and wellbeing, a new foundation is needed to enable space for other questions, possibilities and critical perspectives to emerge.

## Data availability statement

The original contributions presented in the study are included in the article/supplementary material, further inquiries can be directed to the corresponding author.

## Author contributions

BA: Conceptualization, Writing – original draft, Writing – review & editing. SI: Writing – original draft, Writing – review & editing.
